# Targeted exercise against osteoporosis: A systematic review and meta-analysis for optimising bone strength throughout life

**DOI:** 10.1186/1741-7015-8-47

**Published:** 2010-07-21

**Authors:** Riku Nikander, Harri Sievänen, Ari Heinonen, Robin M Daly, Kirsti Uusi-Rasi, Pekka Kannus

**Affiliations:** 1 Department of Medicine, The University of Melbourne, Western Hospital, Melbourne, Australia; 2 Bone Research Group, UKK Institute for Health Promotion Research, Tampere, Finland; 3 Research Department of Tampere University Hospital, Tampere, Finland; 4 Department of Health Sciences, University of Jyväskylä, Jyväskylä, Finland; 5 Division of Orthopaedics and Traumatology, Medical School, University of Tampere, Tampere, Finland; 6 Department of Trauma, Musculoskeletal Surgery and Rehabilitation, Tampere University Hospital, Tampere, Finland

## Abstract

**Background:**

Exercise is widely recommended to reduce osteoporosis, falls and related fragility fractures, but its effect on whole bone strength has remained inconclusive. The primary purpose of this systematic review and meta-analysis was to evaluate the effects of long-term supervised exercise (≥6 months) on estimates of lower-extremity bone strength from childhood to older age.

**Methods:**

We searched four databases (PubMed, Sport Discus, Physical Education Index, and Embase) up to October 2009 and included 10 randomised controlled trials (RCTs) that assessed the effects of exercise training on whole bone strength. We analysed the results by age groups (childhood, adolescence, and young and older adulthood) and compared the changes to habitually active or sedentary controls. To calculate standardized mean differences (SMD; effect size), we used the follow-up values of bone strength measures adjusted for baseline bone values. An inverse variance-weighted random-effects model was used to pool the results across studies.

**Results:**

Our quality analysis revealed that exercise regimens were heterogeneous; some trials were short in duration and small in sample size, and the weekly training doses varied considerably between trials. We found a small and significant exercise effect among pre- and early pubertal boys [SMD, effect size, 0.17 (95% CI, 0.02-0.32)], but not among pubertal girls [-0.01 (-0.18 to 0.17)], adolescent boys [0.10 (-0.75 to 0.95)], adolescent girls [0.21 (-0.53 to 0.97)], premenopausal women [0.00 (-0.43 to 0.44)] or postmenopausal women [0.00 (-0.15 to 0.15)]. Evidence based on per-protocol analyses of individual trials in children and adolescents indicated that programmes incorporating regular weight-bearing exercise can result in 1% to8% improvements in bone strength at the loaded skeletal sites. In premenopausal women with high exercise compliance, improvements ranging from 0.5% to 2.5% have been reported.

**Conclusions:**

The findings from our meta-analysis of RCTs indicate that exercise can significantly enhance bone strength at loaded sites in children but not in adults. Since few RCTs were conducted to investigate exercise effects on bone strength, there is still a need for further well-designed, long-term RCTs with adequate sample sizes to quantify the effects of exercise on whole bone strength and its structural determinants throughout life.

## Background

Osteoporosis, falls and related fragility fractures represent a serious and global public health problem that is projected to increase as our population ages [1,2]. Currently, it is estimated that 30%-50% of women and 15%-30% of men will suffer an osteoporotic fracture in their lifetime [3]. From a biomechanical perspective, fractures represent a structural failure of the bone whereby the loads applied to the bone exceed its strength [4]. Bone strength depends on a number of interrelated factors, including the amount of bone tissue (size and mass), the structure of bone (spatial distribution, shape and microarchitecture) and the intrinsic properties of the bone material (porosity, matrix mineralization, collagen traits and microdamage) [4,5].

To date, most studies have used areal bone mineral density (BMD) measured by dual energy X-ray absorptiometry (DXA) as a surrogate measure of bone fragility. Despite reports that BMD is a good predictor of population fracture risk [6], current research indicates that up to 80% of all low-trauma fractures occur in individuals who are not osteoporotic but have normal or somewhat reduced BMD, i.e., osteopenia [7-9]. This finding highlights the limitations of DXA to provide accurate measures of BMD or its ability to give relevant information about the major determinants of bone strength, such as the size, shape and structure of bone [9]. For instance, small changes in bone mass distribution, cortical and trabecular structure, and bone geometry that contributes to a greater bone cross-section can lead to large increases in bone strength independent of changes in BMD [7-9].

Although exercise is widely recommended as one of the key preventative strategies to reduce the risk of osteoporosis, falls and fractures, its effects on bone have remained controversial because its potential for improving whole bone strength has not yet been properly assessed [10]. However, advances in noninvasive bone imaging techniques, such as peripheral quantitative computed tomography (pQCT) have made such an evaluation possible [11]. Therefore, the primary objective of this systematic review and meta-analysis was to provide an update on the current knowledge regarding the effects of exercise on estimates of whole bone strength throughout the lifespan. Specifically, we analysed the exercise findings by age groups (childhood, adolescence, young adulthood and older adulthood) and compared the exercise-induced changes to habitually active or sedentary controls. We assessed randomised controlled trials (RCTs) in which exercise duration was 6 months or more and 'bone strength' was estimated from quantitative computed tomography (QCT), pQCT, magnetic resonance imaging (MRI) or DXA-based hip structural analysis (HSA).

The secondary aim was to generate new research hypotheses by discussing the structural basis underlying exercise-induced improvements in bone strength, as well as the characteristics of loading that have been shown to be best associated with structural improvements in bone.

In the first section of this systematic review and meta-analysis, we briefly discuss the key findings from previous intervention trials and meta-analyses that have evaluated the effects of exercise on conventional DXA-measured BMD. We then present the findings from our own meta-analysis on the basis of exercise RCTs measuring bone strength as an outcome.

## Methods

We specify our eligibility criteria, data sources, selection of studies, data extraction, sensitivity, and subgroup analyses *a priori*. The procedure was conducted according to the Cochrane reviewers' handbook.

### Eligibility Criteria

For this meta-analysis, we accepted RCTs with an exercise duration of at least 6 months that used (p)QCT, MRI or DXA HSA to assess whole bone strength. The 6-month follow-up limit was selected because the entire bone remodelling cycle typically takes around 6 months, and thus it is unlikely that any true physiological exercise-induced skeletal changes would occur prior to this period. Examiner blinding or participants' ethnicity were not eligibility requirements. Throughout this paper, we will use a common term 'bone strength' to encompass all specific biomechanical terms of bone strength, such as bone strength index (BSI), stress-strain index (SSI), maximal moment of inertia (Imax), cross-sectional moment of inertia (CSMI) and section moduli (Z). We focused on papers published on the topic in English until October 2009. We included studies from the prepubertal period (≥6 to 10 years of age) to older age (≥50) and analysed the results by age groups (childhood, adolescence, adulthood and older adulthood) to reduce the clinical heterogeneity since the effects of exercise on bone strength is an age- and maturity- dependent phenomenon [12]. For instance, it is hypothesized that growth is a time when exercise may be associated with a marked increase in bone strength. In contrast, adulthood is a period when exercise may help to maintain bone strength, while exercise in older adulthood is most likely to reduce (or attenuate) the natural decline in bone strength.

We included healthy female and male children, and adult women and men not using anti-osteoporotic medication, unless equally distributed between study arms in a given trial. We excluded quasi RCTs, trials for people with disabilities and all animal experimental studies. Intervention exercise or physical activity trials were defined as weight-bearing impact, resistance, endurance training or a combination of these types of training. Habitual recreational activity without any specific intervention or supervised activity known not to affect bone (sham exercise) was accepted as activity for the control participants.

### Information Sources and Search

For this systematic review and meta-analysis, an experienced medical librarian conducted a search of four databases (PubMed, Sport Discus, Physical Education Index, and Embase) using a comprehensive combination of keywords describing exercise: exercise movement techniques, exercise, exercise therapy, physical education and training, sports, physical fitness, physical activity, physical training, exercise training, physical exercise, physical education and motor activity. The keywords describing bone strength included bone structure, bone strength, bone rigidity, bone geometry, bone and bones. The authors RD, AH and RN also used cross-referencing from retrieved articles and conducted a hand search to identify possible missed RCTs in database searches.

### Data Collection Process and Quality Analysis

Two authors (AH and RN) independently reviewed all screened abstracts. Then AH and RN, together with RD, independently evaluated all potential full-text articles. If there was disagreement whether to include a full-text article, all authors evaluated the article to come to a joint consensus. Similarly, the methodological quality was individually assessed by AH and RN together with RD. AH then extracted the data from the original full-text articles, and RN and RD rechecked all relevant data, including all outcome values needed for the analysis. Two RCTs where participants were taking either anti-osteoporotic medication or hormone replacement therapy were included because the study arms were equally distributed. Only exercise arms and non-exercise arms without treatment were analysed in these trials because interaction effects were not reported [13,14]. However, two other exercise trials including multiple but heterogeneous exercise groups in their exercise regimen were treated as independent in our analysis [15,16].

Methodological quality was assessed on the basis of individual trial characteristics using the Cochrane risk of bias assessment tool (Tables 1 and 2) [17]. In general, we concentrated on the following issues: 1) sequence generation, 2) allocation concealment, 3) blinding to group assignment, 4) incomplete outcome data, 5) selective outcome reporting, and 6) other potential sources of systematic bias. Specifically, we were interested in whether randomization was accomplished according to the guidelines (items 1 and 2). Moreover, since bone strength measures can be classified as objective outcomes, we evaluated whether optimal blinding was used to avoid control participants' enthusiasm in increasing their exercise level or whether clear contamination existed between exercisers and controls (item 3). The optimal blinding in children was defined as school rather than individual pupil randomization. In adults and older adults, sham exercise (exercise not affecting bone) rather than normal daily activity was defined as optimal blinding to group assignment. We also evaluated whether intention-to-treat (ITT) rather than a per-protocol approach was used in analyzing the data of original articles, as the issues related to participant withdrawals and possible outliers in measurements (item 4). Likewise, we checked the selective outcome reporting of specific results (item 5). Finally, we evaluated all other potential risk of bias, including whether possible imbalances between baseline variables were considered, exercise dose was adequate (at least three times per week), the sample size was large enough (at least 100 participants) and the follow-up time was long enough (12 months or more) to detect an osteogenic response. In addition, we were interested in whether exercise regimens were similar or heterogeneous between the trials. Each item was classified as low risk if the definition of high quality was fulfilled, high risk if the definition was not fulfilled, and unclear if the information was not available in the original article or it was not obtained from the authors. In cases of possible disagreement between the reviewers, all authors reevaluated the article, and a joint decision was made.

**Table 1:** Characteristics of the evaluated 10 trials investigating exercise effects on bone strength^a^

Reference	Population	Intervention	Site Measured	Measurement Technique
	*Children and Adolescents*			
				
Macdonald et al. [19]	EX: N = 281, CON: N = 129 Age: 10.2 (0.6), boys and girls Duration: 16 months	EX: 1 step: 15 min of physical activity (PA) 5 times per week 2 step: 5-36 jumps per day 4 times per week CON: Two 40-min physical education (PE) classes per week	Distal tibia Tibial midshaft	pQCT
Macdonald et al. [20]	EX: N = 293, CON: N = 117, Age: 10.2 (0.6), boys and girls Duration: 16 months	EX: 1 step: 15 min of PA 5 times per week 2 step: 5-36 jumps per day 4 times per week CON: Two 40-min PE classes per week	Hip	DXA-derived hip structural analysis
MacKelvie et al. [21]	EX: N = 31, CON: N = 33, Age: 10.2 (0.5), boys Duration: 24 months	EX: 10-12 min of high-impact PA 3 times per week CON: 10-min stretch during PE class and a stretch break during class	Hip	DXA-derived hip structural analysis
Petit et al. [22]	EX: N = 87, CON: N = 90, Age: 10.1-10.5 (0.5), girls Duration: 7 months	EX: 10-12 min of high-impact PA 3 times per week CON: 10-min stretch during PE class and stretch break during class	Hip	DXA-derived hip structural analysis
Weeks et al. [23]	EX: N = 52, CON: N = 47 Age: 13.8 (0.4), boys and girls Duration: 8 months	EX: 10-min high-impact PA 2 times per week CON: Warmup and stretching exercises during PE classes 2 times per week	Hip	DXA-derived estimation of cross- sectional moment of inertia (CSMI)
				
	*Adults*			
				
Vainionpaa et al. [24]	EX: N = 39, CON: N = 41, Age: 38 (2), women Duration: 12 months	EX: 60-min workout classes including steps and hops 3 times per week and 10-min daily steps and hops CON: Normal daily activity	Proximal tibia Midfemur	DXA-derived estimation of cross-sectional moment of inertia (CSMI)
	*Older Adults*			
				
Cheng et al. [13]	EX: N = 20, CON: N = 20, Age: 50-57, women Duration: 12 months	EX: Supervised high-impact circuit training session 2 times per week and similar home exercises 4 times per week CON: Normal daily activity	Tibial midshaft, Proximal tibia Midfemur, Proximal femur	QCT-derived maximum moment of inertia (I max)
Karinkanta et al. [15]	EX: N = 112, CON: N = 37, Age: 73 (2) Duration: 12 months	EX: Resistance, balance jumping, and their combination group 3 times per week, including at least 50%-60% of repetition maximum (RM) and jumps CON: Normal daily activity	Distal tibia, tibial shaft Femoral neck	DXA-derived hip structural analysis pQCT derived bone strength index (BSI)
Liu-Ambrose et al. [53]	EX: N = 66, CON: N = 32 Age: 79 (3) Duration: 6 months	EX: Resistance and agility training group 2 times per week including at least 50%-60% of RM and ball games, dance movements CON: stretching	Distal tibia, Tibial shaft	pQCT-derived polar stress strain index (SSI)
Uusi-Rasi et al. [14]	EX: N = 41, CON: N = 41, Age: 53 (2) Duration: 12 months	EX: 20-min multidirectional jumps and steps 3 times per week CON: Normal daily activity	Distal tibia, tibial shaft Femoral neck	DXA-derived bone strength index (BSI) pQCT-derived bone strength index (BSI)

^a ^EX, exercise group; CON, control group; pQCT, peripheral quantitative computed tomography; DXA, dual energy X-ray absorptiometry.

**Table 2:** Results of the methodological quality analysis of individual RCTs

	Sequence generation	Allocation concealment	Blinding to group assignment	Incomplete outcome data	Selective outcome reporting	Other potential sources of bias^a^
*Children and Adolescents*						
						
Macdonald et al. [18]	Low risk	Low risk	Low risk	Low risk	Low risk	Low risk
Macdonald et al. [19]	Low risk	Low risk	Low risk	Low risk	Low risk	Low risk
MacKelvie et al. [20]	Low risk	Unclear	Low risk	High risk^b^	Low risk	High risk^a3^
Petit et al. [21]	Low risk	Unclear	Low risk	Low risk	Low risk	High risk^a4^
Weeks et al. [22]	Unclear	Unclear	High risk^c^	Low risk	Low risk	High risk^a2,a4^
						
*Adults*						
						
Vainionpaa et al. [23]	Low risk	Low risk	High risk^d^	Low risk	Low risk	High risk^a3^
						
*Older Adults*						
						
Cheng et al. [13]	Low risk	Unclear	High risk^d^	High risk^e^	Low risk	High risk^a3^
Karinkanta et al. [15]	Low risk	Low risk	High risk^d^	Low risk	Low risk	Low risk
Liu-Ambrose et al. [53]	Low risk	Low risk	Low risk	Low risk	Low risk	High risk^a2,a4^
Uusi-Rasi et al. [14]	Low risk	Low risk	High risk^d^	Low risk	Low risk	High risk^a3^

^a^Other potential sources of bias included 1. >5% (even though not significant) imbalance in bone baseline variables that was not adjusted for in the statistical analysis, 2. Possible inadequate exercise dose according to general exercise recommendations (<3×/week), 3. Small sample size (<100 participants) in relation to the measurement precision, and 4. Short follow-up time (<12-months).

^b^A clear imbalance between withdrawals of controls and trainees in Tanner Stage 1.

^c^Individual adolescent pupils randomised rather than schools

^d^No supervised sham exercise for controls

^e^No intention-to-treat results reported

### Data Synthesis

In the data synthesis of the meta-analysis, Review Manager software (5.0.16; Thomson ResearchSoft, Carlsbad, CA, USA) was used to calculate the pooled effect estimates for the combinations of single effects of RCTs using an ITT approach if possible. ITT data were replaced with per-protocol data in cases where ITT data were not available [13]. To calculate standardized mean differences (effect size), we used the follow-up values of bone strength measures adjusted for baseline bone values. An effect size around 0.2 was considered a small effect, around 0.5 a medium effect and ≥0.8 a large effect. For all analyses, we used an inverse variance weighted random effects model that incorporates heterogeneity into the model. In multiple comparisons with two or more exercise groups, the number of controls was divided among comparisons to ensure that we counted control participants only once in the meta-analysis. Results were considered to be statistically significant at an alpha level of <0.05. Heterogeneity was calculated using the Cochran's Q-test and an alpha level of <0.10 for statistical significance. In addition, *I*^2 ^was used to examine inconsistency in the study findings. In general, values of <25%, 25%-50%, 50%-75% and >75% were suggestive of low, moderate, high and very high inconsistency.

In the results section of this systematic review and meta-analysis, we present our findings from meta-analysis using forest plots of the standardized mean differences for children and adolescents, young adults, and older adults. We then discuss and summarize the findings in the discussion section. In the final two sections of this review, we discuss the structural basis underlying exercise-induced improvements in bone strength, as well as the characteristics of exercise loading that have been shown to be best associated with structural improvements in bone. For these two sections, pertinent cross-sectional studies were reviewed.

## Results

### Exercise Effects on Bone Strength: A Meta-Analysis of Randomised Controlled Trials

**Figure 1:**
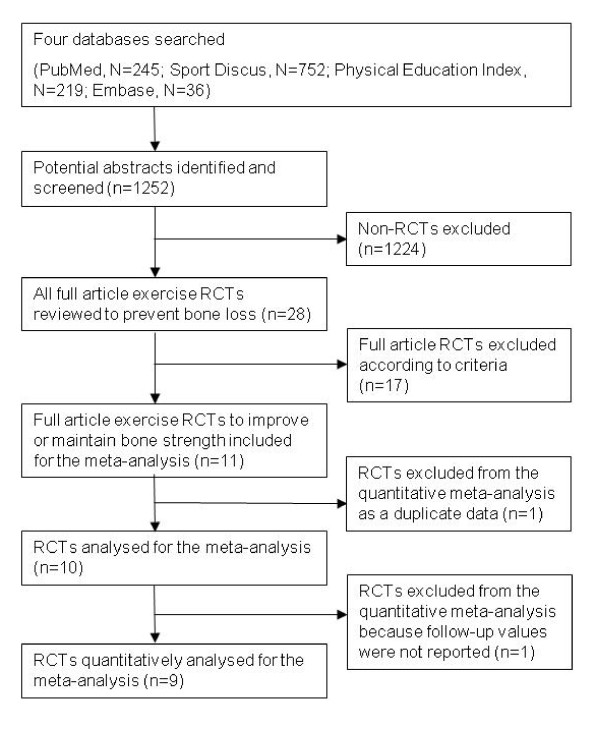
**Flow diagram of the search process of exercise RCTs to improve or maintain bone strength**.

We identified and screened 1252 potential abstracts, of which 1224 were excluded either because they were unrelated to the specific topic, nonrandomised studies or duplicate studies from different databases. We then reviewed the remaining 28 full-text articles of RCTs in which exercise effects on bone were investigated. Seventeen of these 28 trials were excluded because of evaluation of BMD rather than estimates of bone strength. Finally, 11 RCTs investigating exercise effects on bone strength were thoroughly reviewed, one of which was excluded as a duplicate because it used the same sample and a similar outcome as in the investigators' previous trial [18]. Thus, 10 remaining trials were analysed (Figure 1).

From the 10 analysed exercise RCTs that evaluated the effects of different modes of exercise training on estimates of bone strength at different skeletal sites, five were conducted in pre-/early pubertal children and/or adolescents [19-23], one in premenopausal women [24] and four in postmenopausal women [13-16]. To our knowledge, there are no published RCTs examining the effects of exercise on bone strength in adult men. From these 10 trials, the data needed for the effect size estimations were reported in six articles, and the authors provided the data required for three more original articles identified for this review. The findings from the remaining RCT could not be entered into the model because follow-up values were not reported in the original article and could not be obtained from the authors [22]. However, the results were qualitatively analysed, and the findings are discussed in the text. Intervention characteristics of the 10 evaluated studies are summarised in Tables 1 and 2.

Even though we did not exclude articles that may have been biased from our meta-analysis because of the limited number of available RCTs on this topic, the quality evaluation of individual studies revealed several possibilities for systematic bias (Table 2). The major concerns were that 3 of the 10 analysed RCTs were <12 months in duration, and 4 included <100 participants. Moreover, the prescribed type and amount of weekly exercise varied considerably between the different trials. One trial also reported findings on the basis of a per-protocol approach rather than ITT [13]. In addition, some studies included small (albeit not statistically significant) between-group differences in baseline outcome variables despite the randomization. However, we used baseline-adjusted values in our meta-analysis to control for this possible bias. Also, outcome variables describing structural strength varied between the studies, and comparisons were made with controls continuing their habitual activity rather than offering them nonosteogenic (sham) exercise interventions. Other minor concerns were related to the lack of details comparing participants who declined to participate and those who participated in some trials.

### Effects of exercise on bone strength in children and adolescents

#### BMD

Over the past two decades, there have been a number of well-designed exercise RCTs conducted over a period of 6-24 months in prepubertal children and adolescents with BMD as the primary endpoint as reported in a recent review [25]. These studies have shown that exercise programmes incorporating a diverse range of weight-bearing activities can enhance BMC or BMD at loaded skeletal sites such as the femur. A recent systematic review of these randomised and nonrandomised controlled trials indicated that the exercise-induced skeletal gains over 6 months at the femoral neck and lumbar spine ranged from 1% to 6% before puberty and from 0.3% to 2% during adolescence [25].

#### Bone Strength

**Figure 2:**
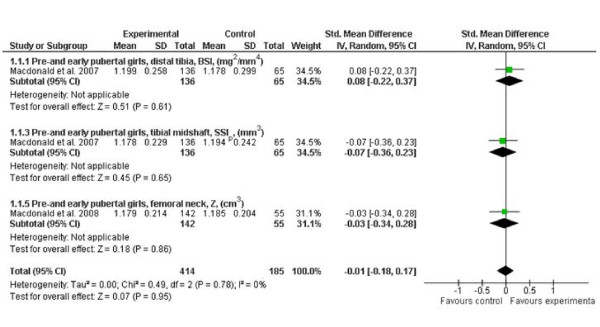
**Effects of exercise on indices of bone strength (standard mean difference, 95% CI) in young girls at the distal tibia, tibial shaft and femoral neck**. The squares and diamonds represent the test values for individual studies and the overall effect, respectively.

**Figure 3:**
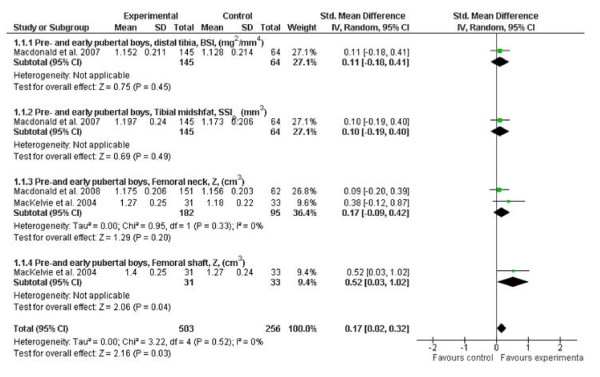
**Effects of exercise on bone strength (standard mean difference, 95% CI) in young boys at the distal tibia, tibial shaft and femoral neck**. The squares and diamonds represent the test values for individual studies and the overall effect, respectively.

Our meta-analysis of published RCTs evaluating exercise effects on bone strength during growth indicate that there was a small but significant effect on the lower extremities in young boys (effect size 0.17; 95% CI, 0.02-0.32), but not in young girls (Figures 2 and 3). Furthermore, when the authors of the original articles conducted per-protocol analyses, which accounted for physiologically meaningful covariates such as weight and height, they reported that weight-bearing exercise can enhance bone strength at the distal tibia in prepubertal but not early pubertal boys [19]. These findings suggest that the response of bone to loading may be maturity-dependent, but further long-term RCTs are still needed to test this hypothesis.

**Figure 4:**
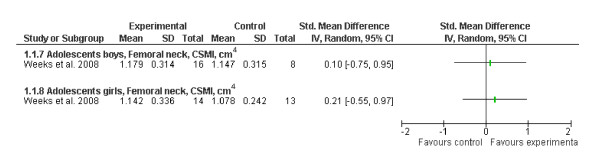
**Effects of exercise on bone strength (standard mean difference, 95% CI) in adolescent boys and girls at the femoral neck**. The squares represent the test values for the individual study.

The results of one RCT were excluded from our meta-analysis because the follow-up bone values were not reported in the original article and could not be obtained from the authors [22]. In this RCT that included pre- and early pubertal girls, the authors reported a 4% exercise effect on femoral neck strength in early pubertal girls. However, our meta-analysis including values of the ITT approach did not find statistically significant exercise effects on femoral neck strength in another RCT including postpubertal adolescent boys or girls. In contrast, per-protocol analysis in this study revealed that there was a trend toward a greater improvement among those who were compliant (19% vs. 11%) in both sexes [23] (Figure 4).

### Effects of exercise on bone strength in adults

#### BMD

In premenopausal women, the findings from several meta-analyses of RCTs examining the effects of different modes of exercise on BMD indicate that resistance training and high-impact weight-bearing exercise, alone or in combination, can produce 1%-2% gains at the lumbar spine and femoral neck [26-29]. Although not all RCTs have reported beneficial effects, the findings from the meta-analyses indicate that high-intensity progressive resistance training appears to be more effective for improving vertebral BMD, whereas high-impact training results in greater gains in femoral neck BMD. Whether exercise has a beneficial effect on BMD in young men is not clear, because few RCTs have been conducted in this population. However, one meta-analysis of randomised and nonrandomised trials incorporating both young and older men reported a site-specific beneficial effect of exercise on BMD in men aged older than 31 years versus those younger than 31 years [30].

#### Bone Strength

**Figure 5:**
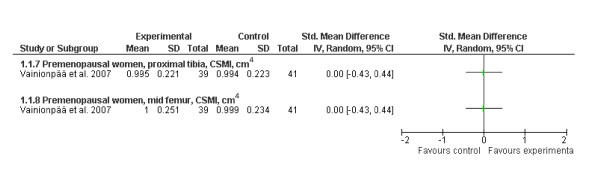
**Effects of exercise on bone strength (standard mean difference, 95% CI) in premenopausal women at the proximal tibia and femoral midshaft**.

With regard to the effects of exercise on bone strength during early adulthood, only one RCT was recently conducted in premenopausal women [24]. Our analysis from this 12-month progressive impact exercise trial with additional home training did not indicate any effect on bone strength at either the proximal tibia or femoral shaft (Figure 5). However, the authors' subgroup analysis of the exercise group revealed that those women who were most compliant (the highest quartile, >66 exercise sessions during the 12 months) had a 0.5%-2.5% greater gain in bone size, cortical thickness and bone strength at the proximal tibia than those who were in the least compliant quartile (<19 sessions).

### Effects of exercise on bone strength in older adults

#### BMD

In postmenopausal women, there are mixed results from several meta-analyses reviewing the effects of aerobic training, weight-bearing impact exercise, resistance training or their combination on BMD [26,28,29,31-36]. In general, the findings from these meta-analyses indicate that lumbar spine BMD can be increased by 1%-2% following resistance training, but findings from the femoral neck have been somewhat contradictory. According to several meta-analyses, endurance training or walking appears to have little or no effect on either femoral neck or lumbar spine BMD [29,31,35,37]. However, a recent meta-analysis reported that mixed-impact loading programs including low- to moderate-impact exercises such as jogging, walking and stair climbing were most effective for preserving BMD at the lumbar spine and femoral neck when combined with resistance training. Interestingly, more demanding high-impact jumping programs without other exercises were ineffective [36]. This suggests that these habitual low-impact exercises combined with other forms of training may be a feasible training mode to preserve BMD in postmenopausal women.

To our knowledge, only one RCT investigating exercise effects on BMD has been conducted in middle-aged and older men alone. In this 12-month trial in healthy community-dwelling men aged 50 to 79 years, a combination of high-intensity progressive resistance training with a diverse range of moderate-impact weight-bearing exercises performed three times per week resulted in a 2% net gain in femoral neck BMD relative to no exercise [38].

#### Bone Strength

**Figure 6:**
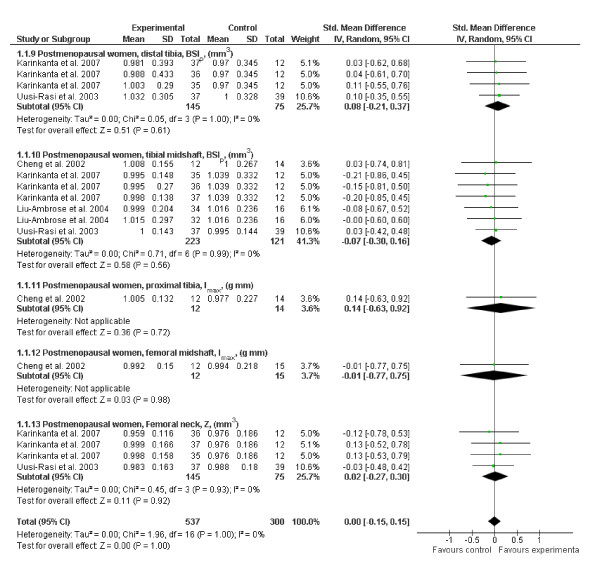
**Effects of exercise on bone strength (standard mean difference, 95% CI) in postmenopausal women at the distal tibia, mid tibia, femoral neck, midfemur and proximal tibia**. The squares and diamonds represent the test values for individual studies and the overall effect, respectively.

Our analysis of the published RCTs examining the exercise effects on bone strength in older women did not find a significant overall effect or any site-specific effects [13-16] (Figure 6). In a recent, yet unpublished, 18-month RCT in middle-aged and older men, high-intensity progressive resistance training combined with moderate-impact weight-bearing exercise increased femoral neck strength by 2% relative to no exercise, but there was no effect of exercise on bone strength at the tibial and femoral midshafts [39]. Consistent with the latter finding, a recent systematic review among postmenopausal women, which included all pQCT studies (RCTs and cross-sectional and prospective studies) in their analysis, concluded that exercise has a positive but modest site-specific effect on bone mass and geometry at the loaded skeletal sites, primarily affecting cortical rather than trabecular bone [40]. The authors also concluded that the mass and geometric changes appear to be largely dependent on continued participation and participants' ability to maintain sufficient exercise intensity.

## Discussion

Despite the well-established benefits of exercise on clinically relevant measures of DXA BMC and BMD, the findings from this systematic review and meta-analysis highlight that there is a need for further well-designed, long-term and adequately powered RCTs before any definitive conclusion can be drawn about the effects of exercise on bone strength. When interpreting the exercise effects on bone strength in children, adolescents and premenopausal women from our meta-analysis, it is important to acknowledge that several of the published papers in each age category were based on data from the same RCTs. Nevertheless, the findings from our meta-analysis of RCTs indicate that there is a small but significant beneficial effect of weight-bearing impact exercise on various estimates of bone strength in young boys at loaded skeletal sites. Furthermore, inspection of the results from the original articles based on the authors' per-protocol analyses indicated that the greatest exercise effect on bone strength tended to occur in prepubertal boys. There was also some evidence for a beneficial effect in adolescent boys and girls who were most compliant with training. Similarly, in middle-aged and older adults, we did not find any significant exercise effects on bone strength, which may partly be explained by the short duration and inadequate power of the few published trials. However, the authors' subgroup analysis again indicated a small effect for premenopausal women who were most compliant with the exercise program [24].

A recent trial investigating exercise effects on BMD reported that small 1%-2% differences in BMD, together with other exercise benefits such as improved muscle function and balance, may reduce fracture incidence by up to 50% [41]. Bone strength is a theoretically meaningful measure of a bone's resistance to fractures. However, measuring bone strength still includes many technical challenges to overcome before it could challenge clinically used DXA BMD and therefore reliably predict fractures. In addition, considering that the precision of pQCT, QCT, MRI, and DXA-related HSA is typically around 2%-3% for estimating bone strength, it is likely that long-term exercise trials (at least 24 months) are needed to accurately quantify the effects of exercise on whole bone strength. Moreover, scanning of bone cross-sections includes other challenges such as defining comparable measurement locations, particularly when performing repeated measures in growing children.

In addition to the above-mentioned challenges, there are a number of other possible reasons why our meta-analysis indicated only small, albeit significant, effects of exercise on bone strength in young children (boys) and no exercise effects in adolescent boys or girls and adult women. Withdrawal of participants and exercise compliance are common problems associated with long-term exercise trials, and thus it is likely that many of the individual trials were not adequately powered to detect potential significant exercise-related benefits on bone strength. It is important that future studies carefully consider the sample size necessary to detect any potential between-group differences. Furthermore, there was considerable variability in the type and dose of exercise prescribed amongst the different intervention trials, all of which may account for the marked variability in the skeletal response to training.

Overall, it is clear from this review that further adequately powered and long-term (>2 years) intervention trials are needed, particularly in adults, because changes in bone structural properties are reportedly small throughout adult life [42].

### Structural Basis Underlying Exercise Gains in Bone Strength

Bones can adapt their strength to increased loads through surface-specific changes on the periosteal or endosteal surface either independently or in combination. For example, loading can increase cortical thickness through periosteal apposition, resulting in an increase in bone size and/or via the addition or reduced resorption of bone on the endocortical surface [43].

**Figure 7:**
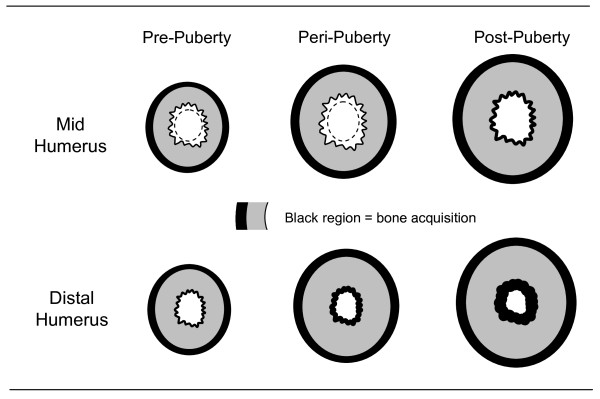
**The changes in cortical bone structure in response to exercise at the mid- and distal humerus in female tennis players**. The increase in cortical area in the prepubertal players was the result of greater periosteal (outer bone surface) than endocortical (inner bone surface) expansion at the midhumerus, but greater periosteal expansion alone at the distal humerus. During the peri- to postpubertal years, loading resulted in both periosteal expansion and endocortical contraction at both sites (adapted from Bass et al. [44]).

During growth, there is evidence that the surface-specific responses to loading are sex-specific and maturity-dependent. For instance, in a study using MRI to compare bone structural differences between the playing and nonplaying arms of young female tennis players, Bass *et al. *[44] reported that exercise-induced gains in humeral bone strength in players prior to puberty were due to periosteal apposition, whereas the predominant effect after puberty was endocortical apposition (Figure 7). Using the same methodology, similar results were recently observed in pre-, peri- and postpubertal boys, but the periosteal gains in prepubertal boys were nearly double those observed in girls and their continued training into peripuberty resulted in further gains in bone size (periosteal apposition) [45]. In both studies, there was heterogeneity in the response to loading at the endocortical surface along the length of the bone. For example, in the prepubertal players, loading resulted in increased expansion at the midhumerus but no change or endocortical apposition at the distal humerus [44-47]. This highlights that there are regional differences in bone adaptation within localized areas of the bone.

In support of the notion that there are regional adaptations to loading, the findings from a recent 16-month school-based exercise intervention revealed that anterior-posterior bending strength at the tibial shaft increased in the active pre- and early pubertal boys [18]. In addition, a recent cross-sectional study involving a range of young adult athletes participating in different sports and a nonathletic reference group revealed that there were surface-specific differences in bone adaptations at the distal tibia and tibial shaft amongst the different types of athletes. Adult athletes involved in impact-type sports had thicker cortices at the distal tibia and tibial shaft, but larger cross-sectional area (bone size) only at the tibial shaft. This suggests that the cortex at the distal tibia may thicken from the endosteum, whereas at the tibial shaft the predominant response was periosteal apposition [48,49].

In older adults, the results from cross-sectional studies indicate that exercise can enhance cortical thickness and bone strength at loaded sites, which appears to be largely due to an increase in the cross-sectional size of the bone (periosteal apposition) [50-55]. However, these findings are not supported by the limited data from exercise intervention trials [56]. In an early non-RCT using pQCT to assess bone geometric changes at the radius in response to a 6-month upper limb loading program in postmenopausal women, Adami *et al. *[57] reported a significant training effect on cortical bone area (3%) and cortical BMC (3%), but a decrease in trabecular BMC (-3%). The authors speculated that increased loading resulted in reshaping of the bone cross-section (periosteal expansion) and a redistribution of bone minerals from the trabecular to cortical component (e.g., corticalisation of the trabecular tissue). Similarly, the findings from a 12-month multidirectional jumping intervention in postmenopausal women revealed that exercise seemed to improve bone strength at the distal tibia by increasing the ratio of cortical to total area [14]. There was no effect of exercise on total area, which suggests that exercise reduced endocortical bone loss.

While some RCTs in older women conducted over 6 to 12 months have failed to observe any significant effect of resistance or impact training on bone structural properties at the tibia or radius [13,16], some beneficial effects on cortical volumetric BMD at both midshafts were observed [16]. Moreover, the results from twin pairs aged 50-74 years who had been discordant for physical activity for at least 30 years indicated that bone strength increased by 18% at the distal tibia, which appeared to be due to an increase in trabecular bone density rather than bone size [51]. At the tibial shaft, however, cortical area and bone strength were 8% and 20% greater in the active compared with inactive twin members, respectively. This was predominantly due to reduced endocortical resorption and not periosteal apposition [51].

On the basis of these findings and other literature, it appears that exercise-induced improvements in bone strength in older adults are likely due to reduced endocortical bone loss and/or increased tissue density rather than an increase in bone size (periosteal apposition) [44]. As noted above, the latter mechanism is typical for growing bones, which have a greater potential for exercise-induced changes in bone size and strength than their mature counterparts [12,44-47]. However, this phenomenon appears to differ for adults; thus, further long-term RCTs in all age groups are needed to substantiate these observations.

Trabecular bone architecture can also adapt to increased loading, but owing to the limited resolution of most current imaging techniques, the effects of exercise on the thickness, number, separation and orientation of trabecular elements in human bones are not well known. However, the findings from two small studies using high-resolution MRI to assess bone microarchitecture revealed that trabecular bone volume at the proximal tibia, distal femur, or both were greater in high-level athletes (gymnasts and Olympic fencers) relative to controls, which was due to an increase in trabecular number and not trabecular thickness [58,59].

### Loading Characteristics to Best Improve Bone Strength

There is considerable interest in defining the optimal dose(s) and characteristics of loading to best improve bone strength (i.e., optimal loading type and programme) so that precise exercise prescription guidelines can be developed. Extensive research using animal models has shown that the skeleton's response to loading is regulated by a number of different loading characteristics, including the magnitude, rate, distribution (pattern) and number of loading cycles [60-63].

Consistent with these findings, data from the available cross-sectional studies and limited intervention trials in children that have used (p)QCT or MRI to characterise changes in bone structure and strength indicate that the most effective programmes (or loading characteristics) are those that incorporate a combination of moderate- to high-impact weight-bearing activities that are variable in nature (i.e., multidirectional) and applied rapidly [11]. Specifically, the most successful exercise programmes appear to be those that incorporate a diverse range of weight-bearing activities (e.g., skipping, dancing, jumping, and hopping) ranging in magnitude from three to nine times body weight and which are performed three to five times per week, preferably on a daily basis, for 10-45 minutes per session [19,64,65].

In middle-aged and older adults, the optimal type and dose of exercise needed to enhance bone geometry and strength is less definitive. The general consensus from recent intervention trials and meta-analyses with BMD as the primary outcome is that low- to moderate-impact weight-bearing exercise in combination with progressive resistance and/or agility training tends to be the most effective for improving hip and spine BMD (or preventing bone loss) and functional ability in both older men and women [15,36,38]. However, further work is still needed to define the specific types of exercises and the associated loads related to these activities that can be safely performed by older adults at varying levels of physical function and fracture risk. While certain moderate- to high-impact exercises are likely to be contraindicated for older adults at high risk of fracture, it is reassuring that few if any adverse effects have been reported from exercise intervention trials that have been conducted in children or in middle-aged or older adults.

**Figure 8:**
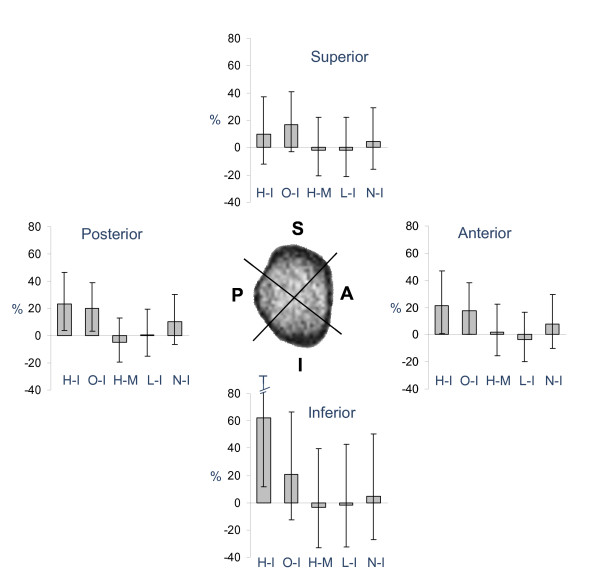
**Athletes representing high-impact (H-I) and odd-impact (O-I) type of exercise loadings clearly have thicker cortices than their sedentary counterparts at the femoral neck**. In a recent cross-sectional study [66], athletes in high-impact sports had 60% thicker inferior cortex; however, athletes representing odd-impact sports had 20% thicker cortex uniformly around the femoral neck, whereas athletes in high-magnitude (H-M), low-impact (L-I), and nonimpact (N-I) sports did not have thicker cortices than their nonathletic counterparts (top) (adapted from Nikander et al. [66]).

On the basis of the available evidence, it is clear that we must await the results of further long-term RCTs before specific exercise prescription guidelines can be developed to maximise bone strength, particularly at the clinically relevant hip and spine. However, a recent cross-sectional study [66] in young adult athletes provided a unique insight into the effects of different modes of loading on femoral neck bone geometry and strength. In this study, MRI was used to examine differences in cortical bone structure and strength in female athletes categorized into five distinct loading groups: high-magnitude vertical impact (volleyball, triple jump, hurdling, and high jump), moderate-magnitude impacts from rapidly varying odd directions (soccer and racket sports), high-magnitude muscle forces (powerlifting), repetitive low impact (running), and repetitive nonimpact (swimming). In comparison to the nonathletic reference group, the authors found that cortical area and bone strength at the femoral neck, but not total cross-sectional area or diameter, were ~15% to 30% greater in athletes representing both the high-impact and odd-impact exercise loading. Further regional analysis examining the cortical thickness at the inferior, superior, anterior and posterior regions of the femoral neck revealed that compared with the reference group, the inferior cortex was ~60% greater in the high-impact group, and the anterior and posterior cortices were 20% greater in both the high-impact and odd-impact loading groups (Figure 8). At the superior cortex, a region subjected to substantial compressive loads from a sideways fall onto the hip [67-69], there was also a trend for a thicker cortex (~15%) in the odd-impact group. These findings suggest that exercise regimes comprising moderate-magnitude impacts from varying, odd directions may represent the optimal mode to enhance bone structure and strength at the clinically important femoral neck [66].

## Conclusion and Future Directions

The benefits of exercise on the clinically relevant DXA measures of BMC and BMD are well established. However, the findings from our systematic review and meta-analyses of published exercise RCTs using (p)QCT, MRI or DXA HSA indicated a small but significant exercise effect on bone strength in young boys but not young girls, adolescent boys or girls or middle-aged and older adults. On the other hand, we acknowledge that there are few long-term, adequately powered RCTs that have investigated exercise effects on bone strength. The available interventions incorporated a variety of different exercise programmes with relatively small sample sizes, short follow-up periods, varied types and doses of training and measurements of different skeletal sites using different imaging techniques. Therefore, we think that it is premature to draw any definitive conclusions with regard to the effects of exercise on bone strength.

Despite the above-noted limitations, the current data from several intervention trials in children indicate that programmes incorporating a diverse range of weight-bearing impact activities can enhance the mass, structure and strength of bone, particularly in boys during the prepubertal years. In middle-aged and older adults, the evidence for a beneficial effect of training on bone strength was less definitive due to the limited number and short average duration of the available RCTs. However, epidemiological evidence suggests that moderate to vigorous physical activity performed three to four times per week is associated with considerably lower incidence of fragility fractures in both women and men [70,71]. In addition, findings from cross-sectional studies of adult athletes suggest that regular exercise for many years has the potential to substantially improve bone strength. The findings from these studies also suggest that exercise regimens that include moderate- to high-magnitude impacts from varying loading directions (high- and odd-impact exercise) may represent the optimal mode to enhance bone structure and strength.

Because whole bone strength is an important determinant of fracture risk, there is still a need for further well-designed, long-term RCTs with an adequate sample size to quantify the effects of exercise on bone strength and its determinants. It is also important to better clarify whether there is an optimal mode and dose of exercise needed to optimise bone strength, particularly at the clinically relevant sites such as the femoral neck and lumbar spine.

Further detailed studies are also needed to characterise the material and structural changes underpinning any exercise-induced gains in bone strength, including region-specific adaptations in the distribution of cortical bone and changes in trabecular microarchitecture. From a clinical perspective, these adaptive processes are important because even small changes in bone geometry and structure can significantly improve bone strength. At present, the available data from intervention trials indicate that BMD and geometry adaptations to loading vary by age, skeletal site and sex. Prior to and early in puberty, the adaptation in cortical bone geometry to loading at diaphyseal sites appears to be mainly due to periosteal apposition [11]. In contrast, at distal skeletal sites containing predominantly trabecular bone, exercise appears to increase tissue density, perhaps due to an increase in trabecular number or thickness or endocortical bone apposition [11]. In adults, the limited data from intervention trials suggest that any increase in bone strength is due largely to increased tissue density, reduced endocortical bone loss, or both, rather than an increase in bone size (periosteal apposition) [56].

Given the importance of cortical thickness and bone size to fracture risk, these findings indicate that the growing years may represent the most opportune time to enhance bone strength and reduce fracture risk. However, we must await the outcome of long-term trials before any final conclusions can be made with regard to whether exercise-induced changes in bone structure and strength translate into a reduction in fracture risk later in life.

## Funding

The funding from the Academy of Finland, Finnish Cultural Foundation, Medical Fund of the Pirkanmaa Hospital District, Finnish Ministry of Education, National Graduate School for Musculoskeletal Disorders and Biomaterials, and the Päivikki and Sakari Sohlberg Foundation is greatly appreciated. Associate Professor Robin Daly is supported by a National Health and Medical Research Council (NHMRC) Career Development Award (ID 425849).

## Competing interests

RN is a shareholder and has a decision-making position in the Devisys Company, which manufactures slip protection devices for shoes.

## Authors' contributions

RN, HS, AH, RD, KUR and PK contributed to the design, data collection, analyses, and manuscript writing of this study.

## Pre-publication history

The pre-publication history for this paper can be accessed here:

http://www.biomedcentral.com/1741-7015/8/47/prepub
